# Data set for renal sinus fat volume and visceral adipose tissue volume on computed tomography

**DOI:** 10.1016/j.dib.2016.04.027

**Published:** 2016-04-19

**Authors:** Yoko Murakami, Yukihiro Nagatani, Masashi Takahashi, Mitsuru Ikeda, Itsuko Miyazawa, Katsutaro Morino, Takayoshi Ohkubo, Hiroshi Maegawa, Norihisa Nitta, Hiroshi Sakai, Hiromitsu Nota, Noritoshi Ushio, Kiyoshi Murata

**Affiliations:** a Department of Radiology, Shiga University of Medical Science, Seta-tsukinowa-cho, Otsu, Shiga 520-2192, Japan; b Department of Radiological Technology, Nagoya University School of Health Science, 1-20 Daikominami 1-Chome, Higashi-ku, Nagoya 461-8673, Japan; c Department of Internal Medicine, Division of Endocrinology and Metabolism Medicine, Shiga University of Medical Science, Seta-tsukinowa-cho, Otsu, Shiga 520-2192, Japan; d Department of Hygiene and Public Health, Teikyo University School of Medicine, 2-11-1 Kaga, Itabashi-ku, Tokyo 173-8605, Japan; e Department of Internal Medicine, Division of Cardiovascular Medicine, Shiga University of Medical Science, Seta-tsukinowa-cho, Otsu, Shiga 520-2192, Japan; f Department of Cardiovascular Surgery, Shiga University of Medical Science, Seta-tsukinowa-cho, Otsu, Shiga 520-2192, Japan

**Keywords:** Renal sinus fat, Coronary calcification, Atherosclerosis, Visceral adipose tissue, Computed tomography

## Abstract

Renal sinus fat is partially characteristic of peri-vascular adipose tissue, however, RSF volume (RSFV) is associated with visceral adipose tissue (VATV). Therefore, the ratio of RSFV to VATV (RSFV/VATV ratio) can distinguish the importance of RSF as an extension of VAT versus its perivascular effects. We assessed the association of RSFV/VATV ratio with coronary artery calcification score (CACS) in 189 patients with suspected coronary artery disease. RSFV of the right kidney and VATV were quantified by using image data of unenhanced abdominal CT. CACS were measured on unenhanced ECG-gated CT images. This article contains data on explanatory scheme of how to measure RSFV on unenhanced abdominal CT, CT indication and exclusion criteria of study population, sex-adjusted association between RSFV with risk factors of coronary vascular diseases and metabolic indices, multivariate linear regression analysis with CACS as the dependent variable in the total study population. The data are supplemental to our original research article describing detailed association between RSFV/VATV ratio and CACS including sub-groups analyses classified by the age of 70 “Renal sinus fat volume on computed tomography in middle-aged patients at risk for cardiovascular disease and its association with coronary artery calcification” Murakami et al. [1].

## Specifications Table

**Table:** 

Subject area	*Biology*
More specific subject area	*Pathogenicity of human adipose tissue*
Type of data	*Table, computed tomography image and figure*
How data was acquired	*Computed tomography scan (Aquilion One; Toshiba Medical Systems, Otawara Japan)*
Data format	*Analyzed*
Experimental factors	*Patients suspected of coronary artery disease*
Experimental features	*VTAV, RSFV, RSFV/VATV ratio, CACS, conventional risk actors of CVD and metabolic indices*
Data source location	*Shiga, Japan*
Data accessibility	*Data are with this article*

## Value of the data

● Could suggest a surrogate evaluator of new treatments of strategy for prevention of atherosclerotic progression in obese cases or metabolic syndrome without chronic kidney disease.
● May facilitate some researchers to quantify renal sinus fat partially characteristic of peri-vascular adipose tissue.
● May stimulate further researches on the clinical significance and utility of RSFV/VATV ratio as prognostic indicators of cardiovascular diseases.
● May provide a crucial clue of how focal peri-vascular adipose tissue deposition play a role on progression in regional atherosclerotic change in further detailed evaluations.

## 1. Data

The data presented in this article include renal sinus fat (RSFV) and visceral adipose tissue (VATV) of 712 patients who underwent coronary CT – angiography between August 2011 and March 2012, measured by Computed tomography scan [1].

## 2 Experimental design, materials and methods

### 2.1 Study subjects

**Fig. 1:**
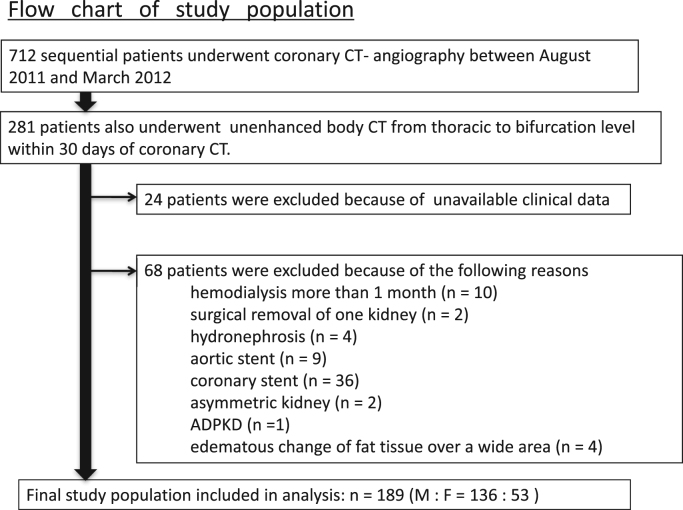
Flow chart of study population ADPKD, autosomal dominant polycystic kidney disease.

Initial enrollment consists in consecutive 712 patients who underwent 320-row ECG-gated computed tomography coronary angiography (CTCA) (Aquilion ONE, Toshiba Medical Systems, Otawara, Japan) by various CT indications during a 10-month period (Table 1). Among 281 patients subjected to both unenhanced abdominal CT examination and blood sample examination within 30 days of the CTCA examination date, clinical data was unavailable for 24 patients and 68 patients were excluded for several reasons (Fig. 1). The data included in this article are derived from remaining the 189 patients. The study was approved by the Institutional Review Board of the Shiga University of Medical Science.

### 2.2 Clinical adipose tissue volumetry

**Fig. 2:**
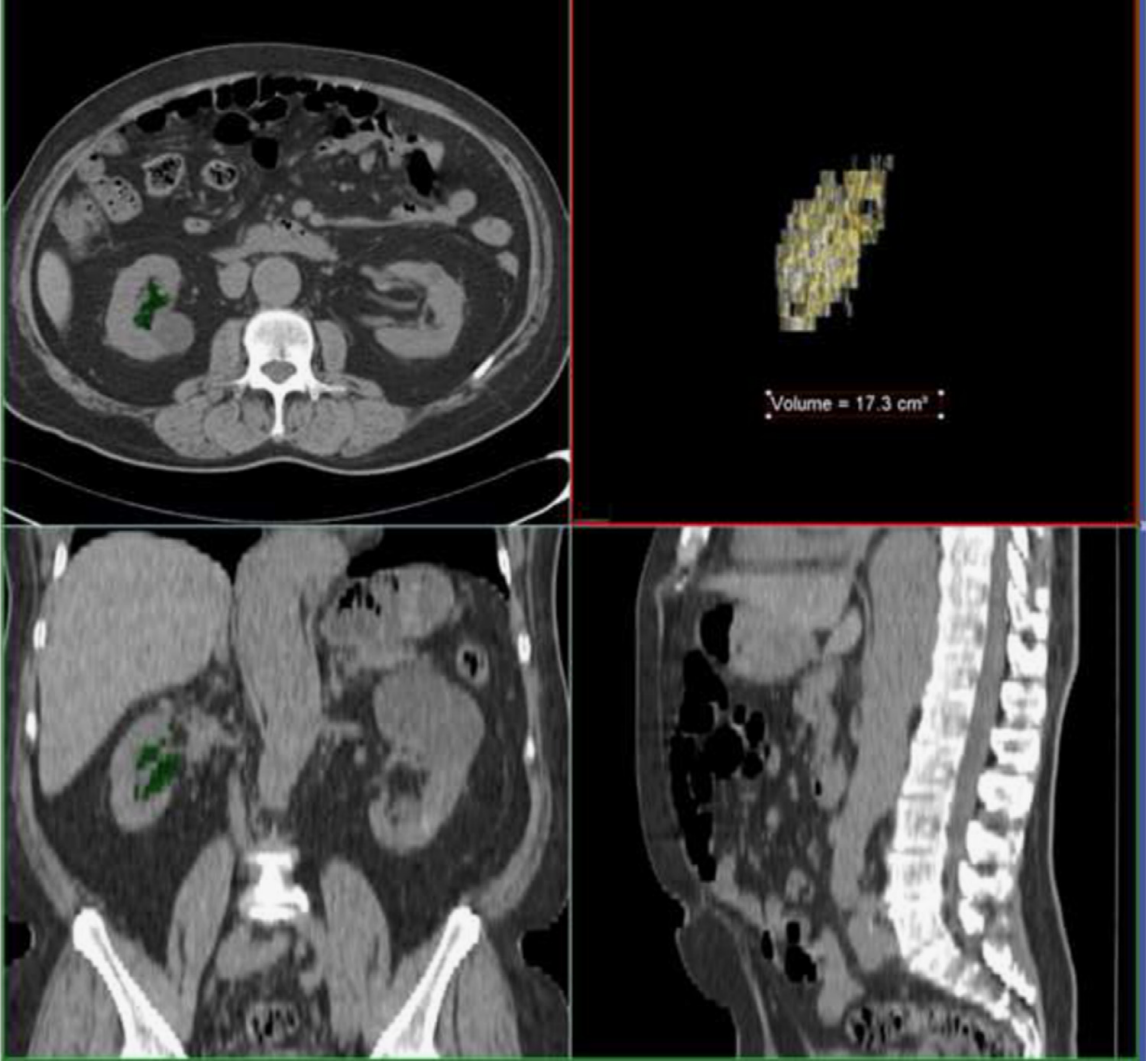
Quantification process of RSFV was modified based on “Framingham Heart Study Renal Sinus Fat Measurement Protocol” (http://hyper.ahajournals.org). In the left images, renal sinus fat is highlighted green, and is demonstrated by a volume rendering technique with the measured value of 17.3 cm^3^ on the right top image.

**Fig. 3:**
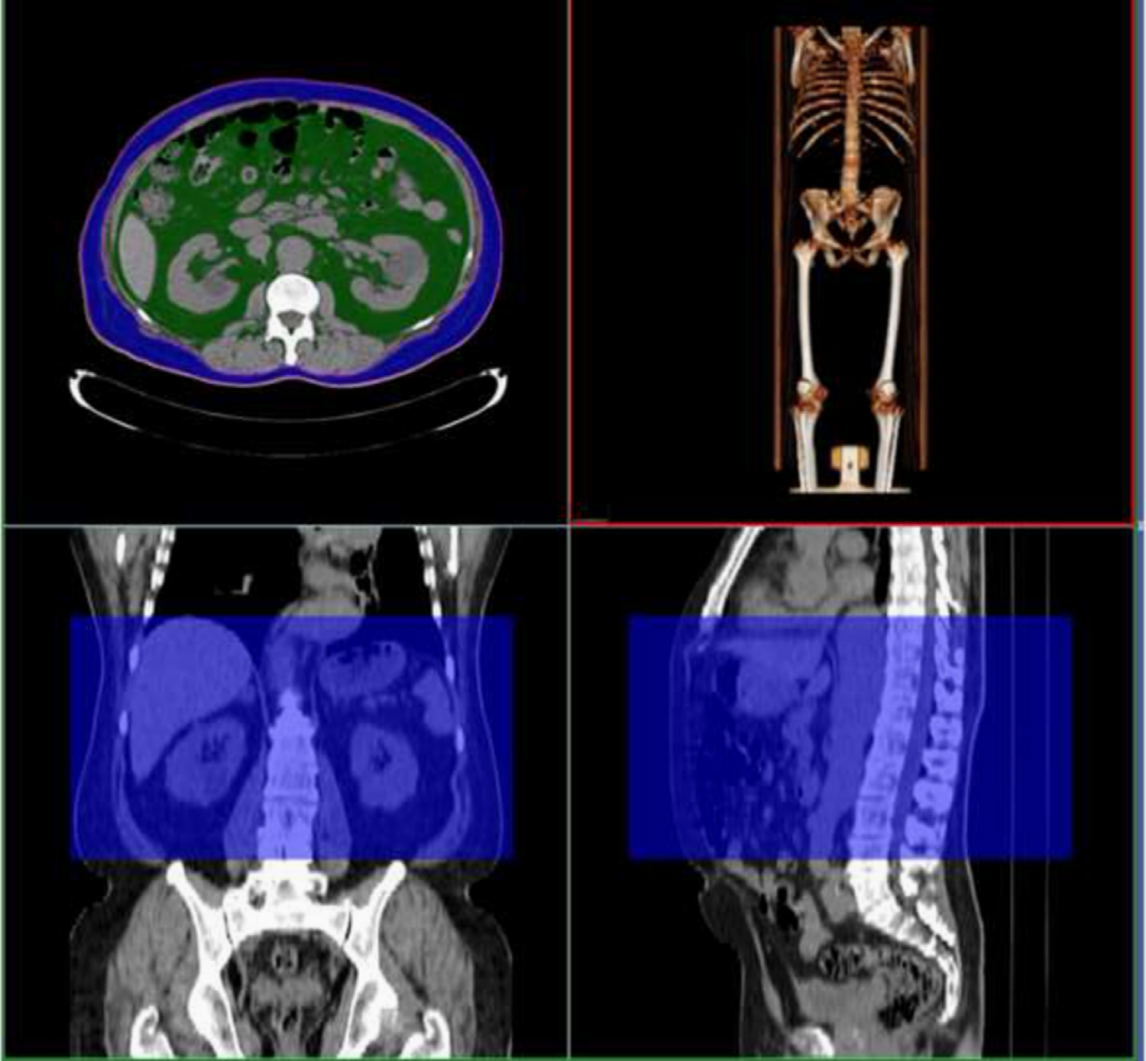
These abdominal CT images are examples of the visceral adipose tissue volume measurement. VATV is highlighted green in the upper left image.

Renal sinus is a region of the kidney in which low pressure venous and lymphatic vessels. VATV and RSFV were measured using a dedicated three-dimensional workstation (Aquarious Intuition, TeraRecon, San Mateo, CA, USA). Measurement in a single median cross-section has been generally adopted for RSF quantification [[2], [3], [4]], however, estimated values based on single cross-sectional measurement may be somehow different from true RSFV due to inevitable measurement error in limited space [5]. According to the previously described protocols [[5], [6]], three to five slices were arbitrarily selected to make slices intervals almost even, and the contour of right kidney was manually traced using polygonal ROI in the selected slices. After an application of contour interpolation technique in the remaining slices, RSFV was automatically measured by the grand total of voxels within CTAV between −195 and −45 HU (Fig. 2). After manual traces of the liver contour in the most cranial slice including the upper liver edge and the inner boundary of the abdominal wall in the most caudal slice including the supra-cristal line followed by contour interpolation technique in the remaining slices, VATV was automatically measured by totaling voxels within the same predefined thresholds as for RSFV [7] (Fig. 3).

### 2.3 Association between RSFV with risk factors of coronary vascular diseases and metabolic indices

RSFV correlated moderately with VATV regardless of the patients׳ age. RSFV showed weak correlations with both HDL cholesterol and kidney in the total study population as well as the middle-aged groups. RSFV correlated weakly with baPWV and hypertension in the middle-aged group, and with pack-years in the total study population. Even after adjusted by sex, these associations of RSFV with risk factors of CVD and metabolic indices were almost similar (Table 2), In addition, RSFV showed a slight but significant positive correlation with systolic blood pressure, which is compatible with the result of past study [3].

### 2.4 Association of RSFV/VATV ratio with CACS

In the total study population, CACS had a weak positive correlation with RSFV/VATV ratio (*r*=0.228, *p*=0.002) and age (*r*=0.316, *p*<0.001). In a multiple linear regression model with gender, RSFV/VATV, hypertension, pack-year, diabetes mellitus, BMI, and kidney volume, only age was associated with CACS (*r*=0.28, *p*<0.01) (Table 3).

## Figures and Tables

**Table 1:** Indication for computed tomography coronary angiography.

	Total	Middle aged group	Elderly group
Chest discomfort	26	17	9
Pre operative coronary screening for aortic disease	38	16	22
Pre operative coronary screening for valvular disease	8	2	6
Pre operation of OPCAB or Evaluation of graft patency post OPCAB	33	15	18
Coronary screening for DM, NFALD or dialysis onset	67	53	14
Others	17	9	8

OPCAB, off-pump coronary artery by-pass; DM, diabetes mellitus; NFALD, Non-alcoholic fatty liver disease; middle-aged group:ages 40–69; elderly group:ages 70–88.

**Table 2:** Partial Pearson׳s correlations (*r*) of RSFV with conventional risk factors of coronary vascular diseases and metabolic indices (sex adjusted).

	Total		Middle-aged group		Elderly group	

BMI	0.41	⁎	0.42	⁎	0.48	⁎
VATV	0.57	⁎	0.61	⁎	0.58	⁎
Kidney volume	0.28	⁎	0.34	⁎	0.27	⁎⁎
eGFR	−0.09		−0.10		−0.11	
Hypertension	0.11		0.19	⁎⁎	0.02	
Systolic blood pressure	0.12		0.19	⁎⁎	−0.05	
Diastolic blood pressure	0.08		0.12		0.00	
baPWV	0.15		0.30	⁎	−0.11	
Pack-year	0.18	⁎⁎	0.12		0.22	
Triglyceride	0.11		0.13		0.05	
HDL cholesterol	−0.23	⁎	−0.28	⁎	−0.15	
LDL cholesterol	−0.08		−0.08		−0.07	
Diabetes mellitus	0.05		−0.002		0.12	

BMI, body mass index; V/S ratio, the ratio of visceral adipose tissue area to subcutaneous adipose tissue area; VATV, visceral adipose tissue volume; eGFR, estimated glomerular filtration rate; baPWV, brachial-ankle pulse wave velocity; HDL, high-density lipoprotein; LDL, low-density lipoprotein.

⁎ *p*<0.01.

⁎⁎ *p*<0.05.

**Table 3:** Multivariate linear regression analysis with coronary calcium score (AU) as the dependent variable in total population.

	Coefficient	SE	*β*	*p* Value	95% CI	
Age	28.0	9.5	0.28	<0.01	9.2	46.9
Gender	−282.2	214.9	−0.13	0.19	−706.8	142.5
Hypertension	−41.6	169.1	−0.02	0.81	−375.7	292.5
Pack-year	0.1	0.1	0.08	0.40	−0.1	0.4
Diabetes mellitus	214.8	157.4	0.11	0.17	−96.1	525.8
Kidney volume	1.0	4.0	0.02	0.81	−6.9	8.9
BMI	0.2	23.6	0.00	0.99	−46.4	46.8
RSFV/VATV ratio	31511.3	39376.7	0.07	0.42	−46302.0	109324.6

CI, confidence interval; SE, standard error; *β*, standardized beta coefficient; RSF, renal sinus fat volume; VATV, visceral adipose tissue volume; BMI, body mass index.
