# Supplementary material for: Data set for renal sinus fat volume and visceral adipose tissue volume on computed tomography
Source: Data Brief. 2016 Apr 19;7:1658–64. doi: 10.1016/j.dib.2016.04.027 (PMC4872677; doi:10.1016/j.dib.2016.04.027)
Supplement: Supplementary file 1 — Supplementary material [file mmc1.pdf]

***Conflict of Interest Form**

Click here to download Conflict of Interest Form: PDF.pdf
